# DIVERSE VOICES OF MENOPAUSE: STUDY RESULTS OF US WOMEN AGES 35 AND OLDER

**DOI:** 10.1093/geroni/igae098.2510

**Published:** 2024-12-31

**Authors:** Jennifer Sauer, Aisha Cozad

**Affiliations:** AARP, Washington, District of Columbia, United States; AARP, Washington, District of Columbia, United States

## Abstract

AARP conducted a bimodal study gauging the impact of menopause symptoms on women and their treatment preferences. The focus groups included 36 women ages 40 and older from a mix of race/ethnicity, education level, and income. The survey of 1,510 U.S. women ages 35 and older included racial/ethnic oversamples of Black/African American (B/AA), Hispanic/Latino (H/L), and Asian American, Native Hawaiian, Pacific Islander women (AANHPI). Three unique multicultural perspectives emerged: 1) symptoms varied by race/ethnicity. For instance, more Black/African American women than other racial/ethnic groups, experience hot flashes (B/AA: 71%; White: 63%; AANHPI: 61%; H/L: 60%); more Hispanic/Latino women than Black/African American or Asian American, Native Hawaiian, Pacific Islander women report mood/emotional swings (H/L: 55%; B/AA: 44%; AANHPI: 36%; White: 49%;); 2) half (49%) all women say symptoms have a negative impact on day-to-day life with more Hispanic/Latino women feeling this way than White or Black/African American women (negative: 63%, 54%, 51%, respectively); 3) more White women than other racial/ethnic groups take psychotropic medications (White: 30%; B/AA: 24%; H/L: 21%; AANHPI: 14%) and more Black/African American, Hispanic/Latino, and Asian American, Native Hawaiian, Pacific Islander women than White women, take vitamins and supplements (B/AA: 57%; H/L: 57%; AANHPI: 57%; White: 47%). HRT use is consistent across racial/ethnic groups. Focus group verbatims, aligned with survey results, provide meaningful insights of the impact of menopause on women.

